# Protective and therapeutic potentials of *Trichinella spiralis* larval antigen in murine induced colitis

**DOI:** 10.1038/s41598-025-13229-3

**Published:** 2025-08-19

**Authors:** Enas A. M. Huseein, Samia S. Alkhalil, Hanan S. M. Farghaly, Haiam M. M. Farrag, Hanaa Y. Bakir, Noha M. Aboulhagag, Samah S. M. Mohamed, Mona Gaber

**Affiliations:** 1 Faculty of Medicine, Department of Parasitology, Assiut University, Assiut, 71515 Egypt; 2 Department of Medical Laboratory Sciences, College of Applied Medical Sciences, Shaqra University, Alquwayiyah, Riyadh Saudi Arabia; 3 Faculty of Medicine, Department of Pharmacology, Assiut University, Assiut, 71515 Egypt; 4 Department of Pathology, Faculty of Medicine, Assiut University, Assiut, 71515 Egypt

**Keywords:** *Trichinella spiralis*, TNBS, Colitis, NO, IL-10 and myeloperoxidase, Immunology, Microbiology, Zoology, Diseases, Gastroenterology, Medical research, Pathogenesis

## Abstract

The intolerable side effects and clinical limitations of current conventional therapies for inflammatory bowel diseases (IBDs), there is a pressing need for alternative treatment options. Helminthes adapt immune responses of their hosts to reduce immune-mediated IBDs. The identification of the mechanism responsible for this beneficial effect on IBDs will provide another feasible approach to treating these diseases. The study was designed to investigate the possible protective and therapeutic role of *Trichinella spiralis (T. spiralis)* crude larval antigen extract in mice challenged with 2,4,6-trinitrobenzene sulfonic acid (TNBS) to induce colitis. Colitis was induced by intra-colonic instillation of TNBS (5 mg/ml in 50% ethanol), preceded or followed by intra-peritoneal (i.p.) administration of a single dose of *T. spiralis* crude larval antigen extract (100 µg/mouse). Colonic damage was assessed macroscopically and microscopically, and the expression of myeloperoxidase (MPO) was evaluated by immunohistochemistry. Colonic interleukin-10 (IL-10) and serum nitric oxide (NO) levels were also measured. Administration of *T. spiralis* crude larval antigen extract before induction of colitis reduced colitis severity as demonstrated by reduced colon weight-to-length ratio, improved macroscopic and microscopic scores, increased colonic IL-10 expression, and diminished colonic MPO protein expression. Moreover, there was a significant negative correlation between serum NO and colonic IL-10 levels. In addition, the preventive potential of *T. spiralis* crude larval antigen extract against TNBS-induced colitis was more prominent than its therapeutic effect. These findings support the hypothesis that *T. spiralis* has both prophylactic and therapeutic potential in inflammatory bowel diseases, which may be via an increase in IL-10 with predominance of its prophylactic role.

## Introduction

Inflammatory bowel diseases (IBDs) are complex chronic disorders, including ulcerative colitis (UC) and Crohn’s disease (CD), which are featured by uncontrolled pathogenic inflammation and damage to the intestinal tissue^1^. The exact etiology of UC remains unclear^2^. Several potential contributing factors, including genetic predisposition and environmental influences, have been proposed. Current theories postulated that colitis results from a dysregulated Th1/Th2 immune response to luminal contents, marked by elevated levels of inflammatory cytokines such as Interleukin-1β (IL-1β) and Tumor Necrosis Factor- (TNF-) and suppressed levels of regulatory Interleukin-10 (IL-10)^3^. This imbalance in intestinal immune homeostasis in patients with IBDs results in a consequent shifting toward the pro-inflammatory side. Thus, the Th1/Th2 balance is important for preventing the development of the disease^4,5^.

IL-10 plays an essential role in the maintenance of intestinal immune homeostasis and plays a critical role in the down-regulation of Th1 responses^6,7^. Several conflicting studies have been published measuring the level of IL-10 in the gut during colitis^8^. However, some studies demonstrated an increase in their level^9,10^. Other studies reported no significant changes at both the protein and mRNA levels^11^. Wang et al. (2015) reported a significant decline in IL-10 levels in the peripheral blood of Wistar rats with TNBS-induced colitis^12^.

TNBS-induced colitis appears to be mediated through a classic delayed-type hypersensitivity reaction by T cells responding to “hapten-modified self-antigen.”^13^. The reaction is formed by the covalent attachment of the hapten, trinitrophenyl to self-proteins. Ethanol is frequently used as a vehicle for TNBS, although it is known to cause local irritation^14^.

According to several related studies, Helminthic infections or worm-derived products protect the host against various immunologically mediated diseases and hypersensitivity disorders by inducing strong anti-inflammatory networks^15^. Therefore, the use of helminthes, which are known to regulate the host’s immune system and prevent excessive inflammatory responses, is being tested as a novel potential treatment strategy for IBDs^16,17^.

Among the different helminthes, *T. spiralis* is unique because all three developmental stages of the parasite develop in the same host (infective muscle larvae, adult, and newborn larvae)^18,19^. During the different phases of parasite growth, *T*. *spiralis* has evolved to suppress inflammatory responses of the immune system to survive in its hosts, and each response is stage-specific^20^. These properties may equip them with the capacity to reduce the severity of inflammatory diseases within their host. However, larval stage of *T. spiralis* is known to produce potent immunomodulatory molecules that modify host inflammatory responses, which is critical for their survival in muscle tissue. These molecules are particularly effective in modulating Th2 responses and promoting anti-inflammatory cytokines like IL-10. The adult worm extract may not exhibit the same immunoregulatory profile, as its primary interaction with the host occurs in the intestinal phase, which involves different immune evasion strategies^21,22^.

*T. spiralis* targeting immune pathways during intestinal inflammation may unveil novel therapeutic approaches for inflammatory bowel diseases (IBDs). Therefore, this study evaluated the efficacy of *T. spiralis* larval antigen extract in both preventive and therapeutic regimens against TNBS-induced colitis in mice, as well as the possible mechanistic basis for its immunomodulatory effects.

## Materials and methods

### Ethical consideration

The experimental protocol was approved by the Ethics Committee of the Faculty of Medicine, Assiut University, Egypt, in accordance with the International Guiding Principles for Biomedical Research Involving Animals as issued by the Council for International Organizations of Medical Sciences (IRB code:17300218). All animal experimental procedures and methods are reported in accordance with ARRIVE guidelines.

### Animals

All tested animals were adult male Swiss albino mice, 20–25 gm weight, were obtained from the animal housing facility, Faculty of Medicine, Assiut University, Egypt. One week prior to experimentation, the animals were maintained under standard conditions of light and temperature in the laboratory and allowed ad labium access to food and water^23^.

### Parasite antigen preparation

The *T. spiralis* strain employed in this study was initially obtained from infected pig diaphragms collected at the El-Bassatine Abattoir in Cairo, Egypt. The parasite was subsequently maintained in the laboratory of the Faculty of Medicine at Assiut University through cyclical passages in Swiss albino mice, following the protocol established by Gamble^24^. *T. spiralis* crude larval antigen was prepared from the encysted larvae using the method of Hassanain et al.. Larvae were repeatedly washed several times by sedimentation in Phosphate-Buffered Saline (PBS) containing phenyl methyl sulphonyl fluoride and sodium azide, kept on ice in PBS, homogenized, followed by sonication. The homogenate was then centrifuged at 4,000 rpm at 4 °C for 30 min. The resulting pellet was discarded, and the supernatant was subjected to a second centrifugation step. Parasite’s antigen was then sterilized by filtration through a 0.22 μm pore membrane filter^25^. The protein concentration of the samples was quantified using Bradford’s assay, standardized to a final concentration of 1 mg/mL, aliquoted, and stored at 20 °C until further use^26^.

### Induction of experimental colitis

Before colitis induction, mice were fasted for 24 h to minimize colonic fecal content while allowing free access to water. Subsequently, the animals were anesthetized via intraperitoneal injection (i.p.) of sodium pentobarbital (50 mg/kg body weight). Subsequently, intracolonic administration of 2,4,6-trinitrobenzenesulfonic acid (TNBS; Sigma-Aldrich, St. Louis, MO, USA). For TNBS delivery, a soft pediatric catheter (6-Fr), tip coated with Vaseline, was inserted 5 cm into the colon, and 0.12 mL of TNBS solution (5 mg/mL in 50% ethanol) was slowly instilled. To ensure proper distribution and prevent leakage, mice were held in a vertical position for 30 s post-instillation, facilitating uniform contact between the TNBS solution and the colonic mucosa^27^.

### Study groups and experimental setup

A total of 50 mice were randomly distributed among five experimental groups, with ten animals in each group (*n* = 10/group).

Group I (Control group): received 0.12 ml of 50% ethanol once by intra-colonic instillation.

Group II (Larval antigen extract group): received a single i.p. dose of *T. spiralis* larval antigen extract (i.p., 100 µg/mouse) without induction of colitis and sacrificed 5 days after antigen inoculation^28^.

Group III (TNBS group): received 0.12 ml of TNBS in 50% ethanol intra-rectally once and were sacrificed 3 days after TNBS instillation^29^.

Group IV: Treatment group (TNBS then larva extract group) subjected to induction of colitis, followed by a single i.p. injection of *T. spiralis* larval antigen extract (100 µg/mouse) on the third day post-colitis and sacrificed 5 days after antigen inoculation^28,29^.

Group V: Protective group (Larva then TNBS group) received a single i.p. injection of *T. spiralis* larval antigen extract (100 µg/mouse) followed by 0.12 ml of TNBS in 50% ethanol solution intra-rectally on the fifth day and sacrificed 3 days after TNBS instillation^28,29^.

### Sample collection

At the end of the study, the animals were sacrificed following final body weight measurements. All mice were euthanised by i.p. injection of a ketamine-xylazine mixture (90 mg/kg body wt. ketamine and 10 mg/kg body wt. xylazine), following American Veterinary Medical Association guidelines^30^. Cardiac puncture was performed to obtain blood samples, which were centrifuged to isolate serum. Serum aliquots were stored at -20 °C for subsequent NO quantification using the Griess reaction^31^. A 4-cm segment of the distal colon (above the anus by 1 cm) was excised and longitudinally incised for: Stool consistency scoring; Macroscopic inflammation assessment; Measurement of colonic edema (wet/dry weight ratio); local IL-10 quantification and histopathological evaluation. Prior to analysis, colonic specimens were gently irrigated with ice-cold 0.9% saline to remove luminal contents. The spleen was also dissected and weighed as an indicator of systemic immune activation^32^.

### Scoring of stool consistency

Fecal consistency was quantitatively evaluated as an indicator of diarrheal activity using standardized criteria adapted from Cooper et al. (Score 0: Normal, well-formed; 1: Mildly soft stool pressure; 2: Very soft stool; Score 3: Watery stool, or severe diarrhea)^33^.

### Assessment of colonic oedema

The colonic weight-to-length ratio (W: L) was used as a secondary marker of disease-associated intestinal wall pathological changes^34^, which were correlated with the intensity of inflammation. The colonic W: L ratio of each mouse was calculated and compared with that of the control group.

### Macroscopic scoring of colonic damage

Macroscopic mucosal damage was assessed by an independent observer utilizing a standardized scale ranging from 0 to 5, defined as follows: 0 (no damage); 1 (localized hyperemia without ulceration); 2 (single ulceration site without inflammation); 3 (single ulceration site with inflammation); 4 (multiple ulceration and inflammation sites, each < 1 cm in size); and 5 (extensive inflammation and ulceration, with lesions ≥ 1 cm)^35^.

### Determination of serum nitrite production

Nitrite, a stable end-product of nitric oxide (NO) auto-oxidation, serves as an indicator of NO production. The nitrite concentration was quantified spectrophotometrically via the Griess reaction, following the method described by Green et al. The Griess reagents consisted of 1% sulfanilamide in 5% phosphoric acid (sulfanilamide solution) and 0.1% N-1-naphthylethylenediamine dihydrochloride (NED solution) in bi-distilled water. A standard curve, which was generated using sodium nitrite and expressed in µmol/L, was prepared concurrently with the sample analyses. Absorbance measurements for both standards and samples were recorded at 550 nm using an Ultrospec Plus UV/Visible spectrophotometer (Pharmacia Biotech, Cambridge, UK)^31^.

### Microscopic evaluation

Freshly excised colonic segments were divided into two portions: one for histopathological evaluation and the other stored at − 80 °C for biochemical analysis. For conventional histopathological assessment, tissue samples were fixed in 10% buffered formalin, embedded in paraffin, sectioned into 5-µm-thick slices, and stained with hematoxylin and eosin (H&E) for light microscopic examination. The severity of colitis was evaluated using the histological scoring system established by Dieleman et al.. Briefly, tissues were graded on a scale of 0–40 based on the following parameters: inflammation severity (0: none; 1: mild; 2: moderate; 3: severe), inflammation extent (0: none; 1: mucosa; 2: mucosa and submucosa; 3: transmural), Crypt damage (0: none; 1: basal one-third damaged; 2: basal two-thirds damaged; 3: only surface epithelium intact; 4: complete crypt and epithelial loss), and Percentage of ulceration/erosion involvement (1: 1–25%; 2: 26–50%; 3: 51–75%; 4: 76–100%). The combined score of the first three parameters (severity, extent, and crypt damage) was multiplied by the percentage involvement factor to derive the final histological score for comparative analysis^36^.

### Immunohistochemistry

Immunohistochemical analysis of myeloperoxidase (MPO) was performed to assess neutrophil infiltration in colonic tissue. The procedure was conducted as follows: Paraffin-embedded tissue Sect. (5 μm thickness) were subjected to de-paraffinization through three xylene washes (5 min each), followed by rehydration in a graded ethanol series. After two washes with phosphate buffer (pH 7.2), antigen retrieval was performed by microwave heating (15 min) in sodium citrate buffer (pH 6.0). Endogenous peroxidase activity was quenched with 3% hydrogen peroxide treatment for 10 min, followed by three 5-minute washes with PBS (1×). Sections were incubated with rabbit polyclonal anti-MPO primary antibody (1:100 dilution; Thermo Scientific, Cat. #RB-373, South San Francisco, CA, USA) at 4 °C for 24 h (50 µL per slide). Detection was achieved using the UltraVision Detection System anti-polyvalent HRP/DAB kit (Thermo Fisher Scientific, Fremont, CA, USA) according to the manufacturer’s protocol. Chromogenic development was assessed using 3,3’-diaminobenzidine (DAB), followed by hematoxylin counterstaining. Appropriate controls were included: tonsillar tissue sections served as positive controls, whereas negative controls were processed with primary antibody omission to assess nonspecific binding. Slides were examined using an Olympus CX41 light microscope (Olympus, Center Valley, PA, USA) equipped with a U-CMAD3 digital camera system. Quantitative analysis was performed by counting MPO-positive cells in 10 randomly selected high-power fields (400× magnification) per section. The mean value of positive cells per field was calculated for each sample to determine neutrophil infiltration levels^37^.

### Estimation of IL-10 concentration in the colon

Previously weighed colon samples from all experimental groups were homogenized in ice-cold phosphate buffer (pH 7.4) using a specify homogenizer type. The homogenates were centrifuged at 10,000 × g for 20 min at 4 °C to obtain cell-free supernatants, which were aliquoted and stored at -20 °C until analysis. IL-10 concentrations in the supernatants were determined using a commercially available enzyme-linked immunosorbent assay (ELISA) kit (Chongqing Biospes Co., Ltd., China) following the manufacturer’s protocol.

### Statistical analysis

The results are presented as mean ± S.E.M. derived from 8 to 10 biological replicates (mice) per experimental group. The normality and homogeneity of variance were verified prior to parametric analysis. For multiple group comparisons, statistical significance was determined using one-way analysis of variance (ANOVA), followed by Dunnett’s post hoc test for controlled comparisons against a reference group^38^. A probability value (*P*) ≤ 0.05 was considered statistically significant for all analyses. Non-parametric correlation analysis was performed using Spearman’s rank correlation coefficient^38^. All statistical computations were executed using Prism software version 5 (GraphPad Software, La Jolla, CA, USA), with two-tailed tests employed throughout the study.

## Results

### Effects of TNBS-induced colitis and *T. spiralis* antigen on body weight

**Fig. 1:**
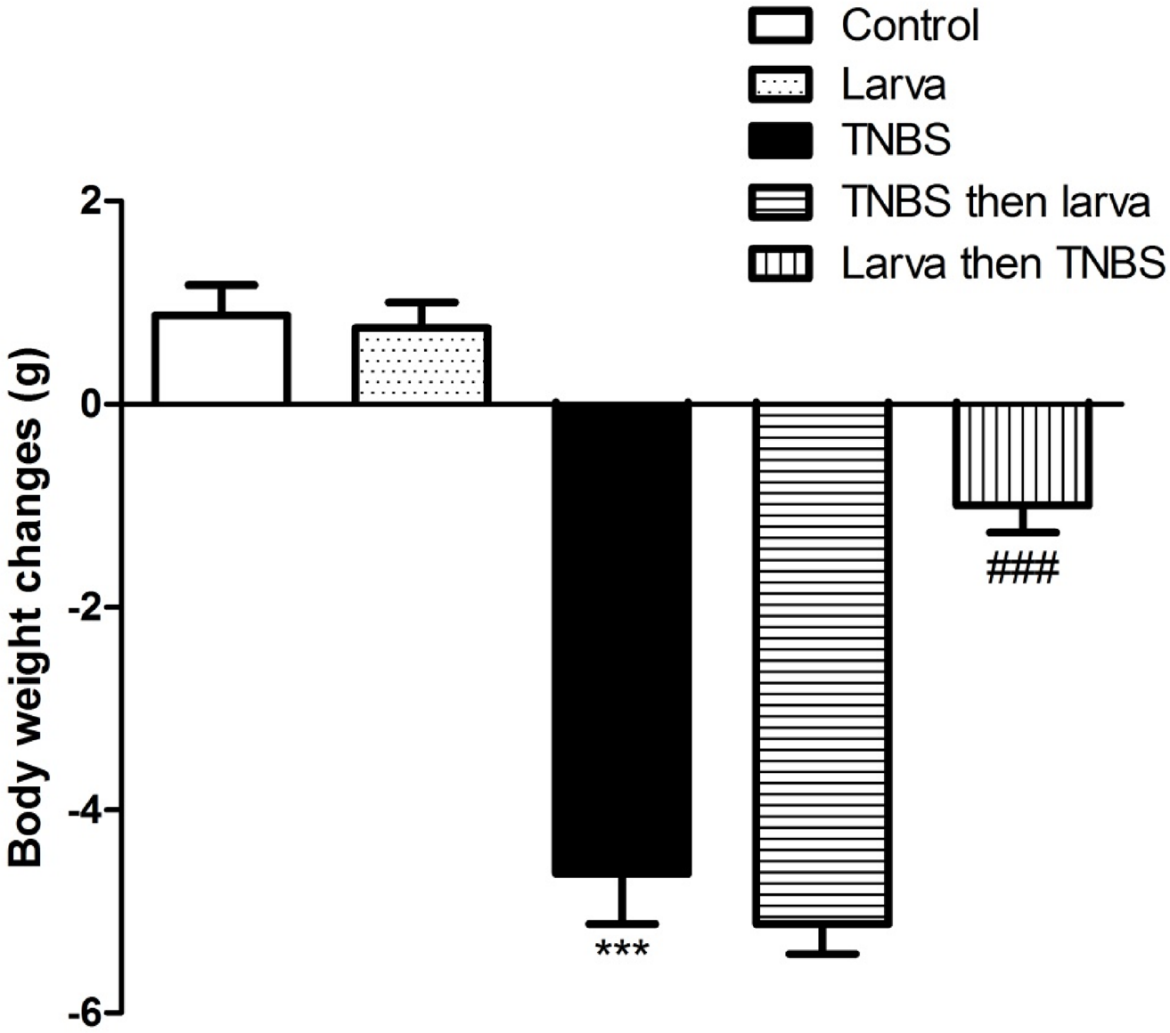
Effects of *T. spiralis* larval antigen on body weight among study groups. Data are expressed as means ± S.E.M.; Significant difference between groups in comparison to negative control expressed as***P < 0.001; while ###*P <* 0.01 indicate difference versus the TNBS-induced colitis group without treatment.

Body weight was improved in both control (G1) and larva extract groups (G2) (0.88 ± 0.3 g, 0.75 ± 0.3 g, respectively). Body weight loss was observed in TNBS-induced colitis (-4.63 ± 0.5 g). The protective group (larva then TNBS) showed a significant reduction in body weight loss compared to the TNBS-induced colitis alone group (*P* < 0.01; “Fig. 1”). However, a body weight drop was still detected in the treatment group (TNBS then larva) (-5.13 ± 0.3 g) (*P* > 0.05; “Fig. 1”).

### Effects of TNBS-induced colitis and *T. spiralis* antigen extract on spleen weight

**Fig. 2:**
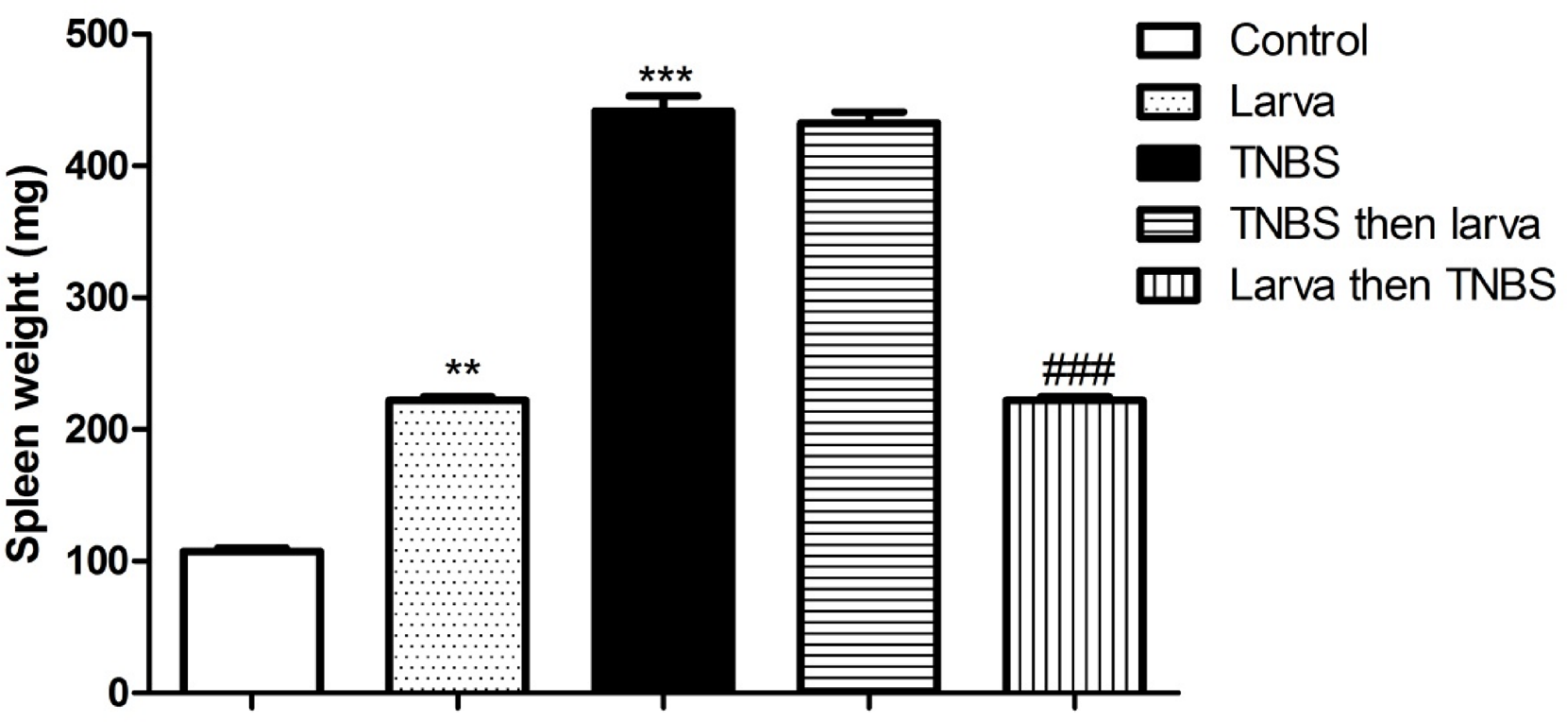
Effects of *T. spiralis* larval antigen on the spleen weight of all studied groups. Data are articulated as means ± S.E.M.; Significant difference between groups in comparison to negative control expressed as**P < 0.01, ***P < 0.001; while ###*P <* 0.01 indicate difference versus the TNBS-induced colitis group without treatment.

*T. spiralis* larval antigen extract increased spleen weight (*P* < 0.01, Fig. 2). The results also showed a significant rise in spleen weight in the TNBS-induced colitis and treatment groups. However, pretreatment with *T. spiralis* larval antigen extract significantly reduced the increase in the spleen weight compared to the control level (*P* < 0.001, “Fig. 2’).

### Effects of TNBS-induced colitis and *T. spiralis* antigen extract on colon weight/length ratios

**Fig. 3:**
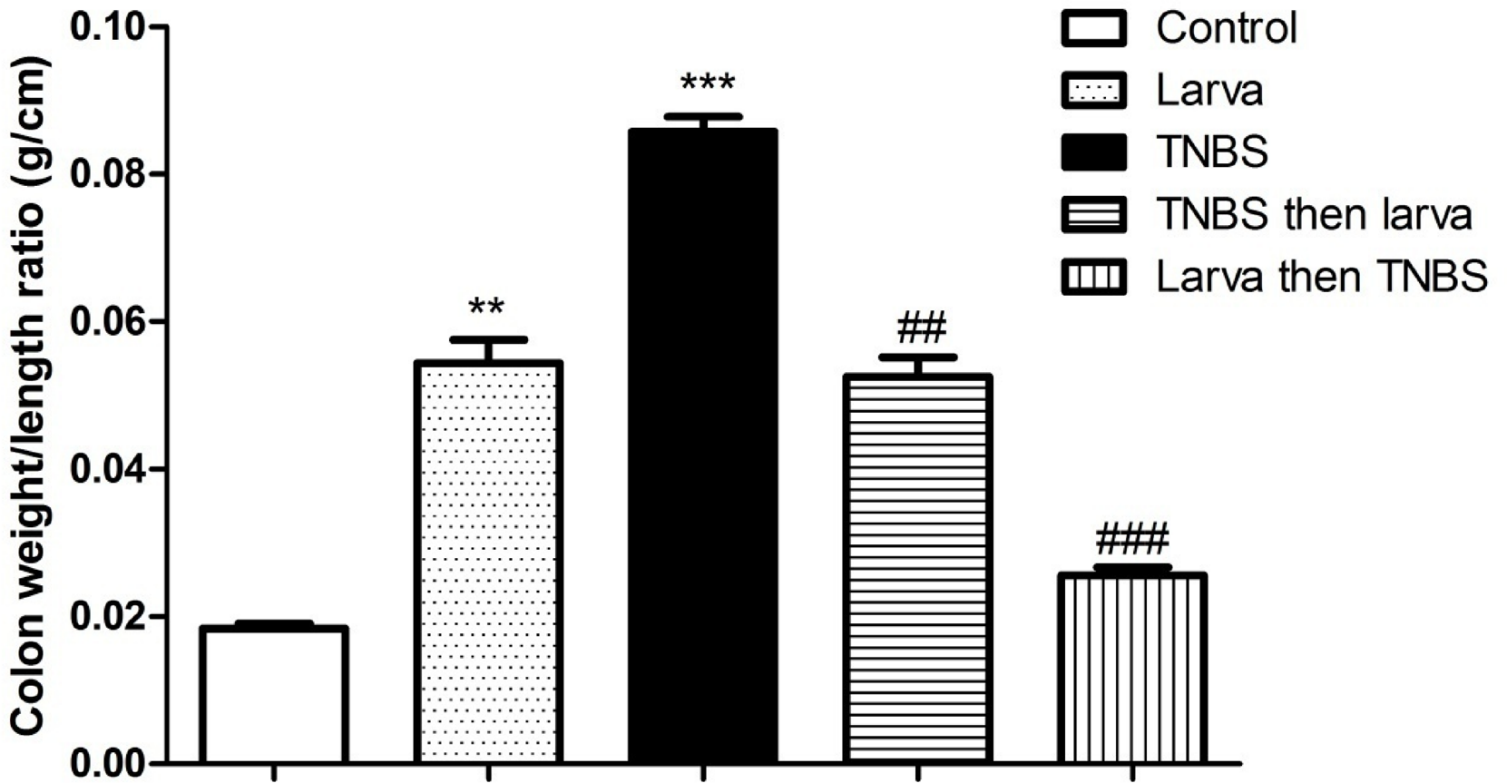
Effects of *T. spiralis* larval antigen on colon weight/length ratio of all studied groups. Data are expressed as means ± S.E.M.; Significant difference between groups in comparison to negative control expressed as **P < 0.01, ***P < 0.001; while ###*P <* 0.01 indicate difference versus the TNBS-induced colitis group without treatment.

The colon W: L ratio was significantly higher in the TNBS-induced colitis group (0.09 ± 0.02 g/cm, Fig. 3) than in the negative control group (0.02 ± 0.01 g/cm, Fig. 3). However, pretreatment with *T. spiralis* larval antigen extract prior to colitis induction significantly reduced this ratio (0.03 ± 0.01 g/cm; *P* < 0.001; Fig. 3). Compared with the TNBS-treated group, the treatment group exhibited a significant reduction in the colon W: L ratio (0.05 ± 0.01 g/cm vs. 0.09 ± 0.02 g/cm; Fig. 3).

### Relationship between changes in colonic IL-10 concentration and serum nitrite level

**Fig. 4:**
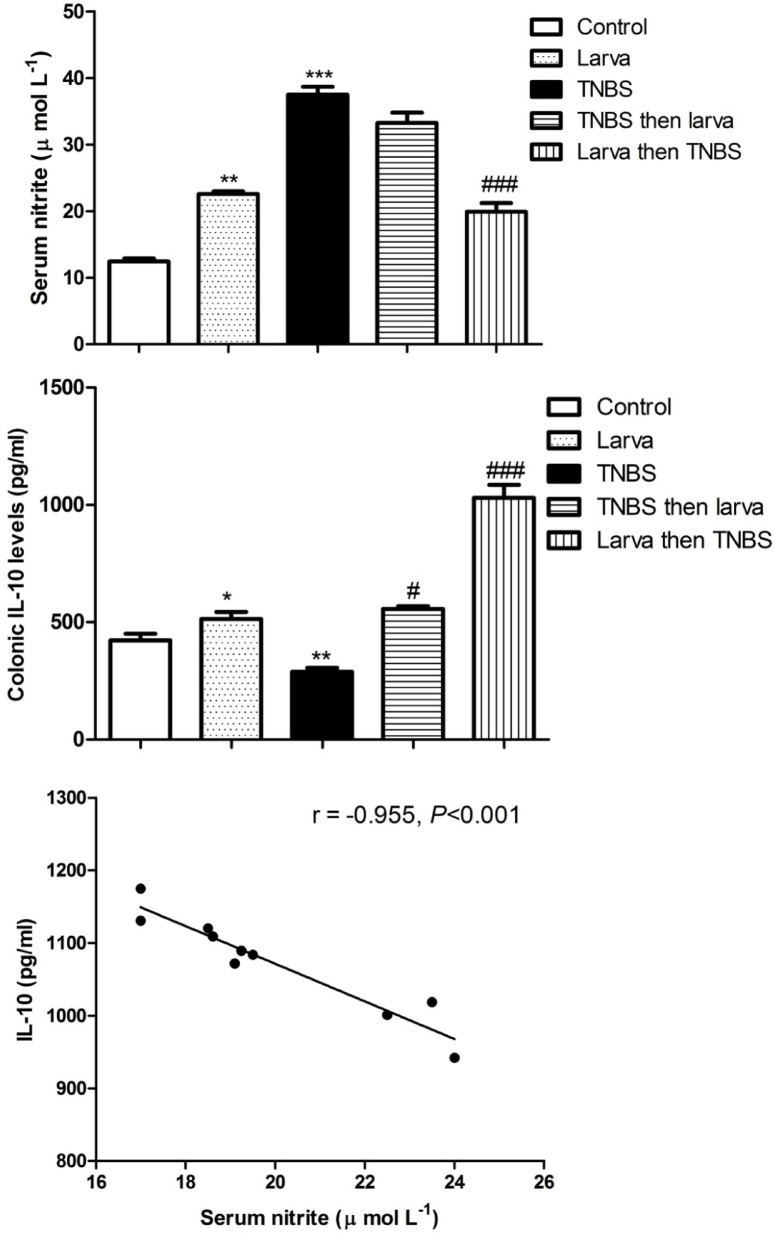
Correlation between changes in colonic IL-10 concentrations and serum nitrite level. Upper panel: Comparison of serum nitrite levels in all studied groups. Middle panel: Comparison of colonic IL-10 levels in all experimental groups. Data were articulated as Mean ± S.E.M. (*n* = 10 mice/group). Significant difference between groups in comparison to negative control expressed as *P < 0.05; **P < 0.01; ***P < 0.001; while ###*P <* 0.01 indicate difference versus the TNBS-induced colitis group without treatment. Data were analyzed by one-way ANOVA, subsequently using Dunnett’s test. Lower panel: Negative correlation between local IL-10 levels in the colon and nitrite serum levels (*r* = − 0.955, *P* < 0.001).

Serum nitrite levels were significantly elevated in both the *T. spiralis* larval extract and TNBS-induced colitis groups. However, pretreatment with *T. spiralis* larval antigen extract prior to colitis induction resulted in a lower nitrite level compared to the TNBS-induced colitis group (Fig. 4, upper panel). IL-10 production was lower in the TNBS-induced colitis group, but the level was reversed when larval antigen extract was administered either before or after TNBS (Fig. 4, middle panel). When the correlation between colonic IL-10 production and serum nitrite level was examined, a negative correlation was observed between serum nitrite and colonic IL-10 levels in the preventive (larva then TNBS) group. (Fig. 4, lower panel).

### Gross appearance of the distal colon among the different groups

**Fig. 5:**
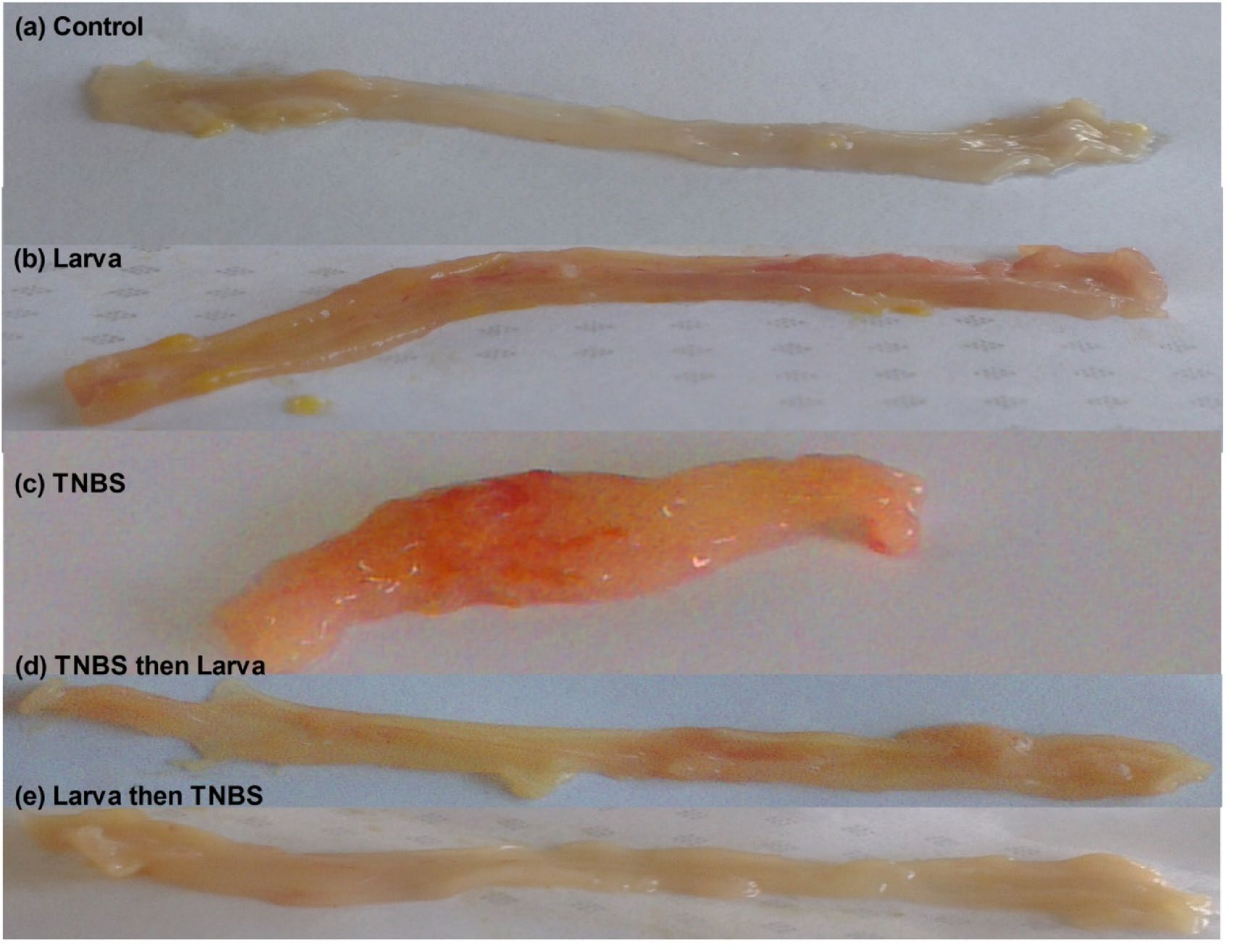
Colonic macroscopic appearance of all studied groups. (**a**) Negative control group; (**b**) Larva control group; (**c**) TNBS-induced colitis without treatment group; (**d**) Treatment (TNBS then larva) group; (**e**) Preventive (Larva then TNBS) group.

As described in Fig. 5a, the vehicle control group exhibited a normal macroscopic appearance, whereas TNBS alone showed colonic mucosal ulceration, oedema, and haemorrhage (Fig. 5c). This gross damage returned to near-normal and normal mucosa in the treatment and protective groups, respectively (Fig. 5d,e) (Table 1).

**Table 1:** Effects of TNBS-induced colitis and *T. spiralis* larval antigen on stool consistency and macroscopic damage in mice*.

Group	*N*	Stool consistency	Macroscopic damage
Control	10	0	0
Larva	10	1–2	1
TNBS	8	3	4
Treatment (TNBS then larva)	8	3	4
Preventive (larva then TNBS)	10	0–1	1–2

*Colonic parameters were quantified in the control, TNBS-induced colitis, larval, treatment (TNBS before larval), and Preventive (larva then TNBS).

### Histopathological evaluation of the distal colon architecture across experimental groups

**Fig. 6:**
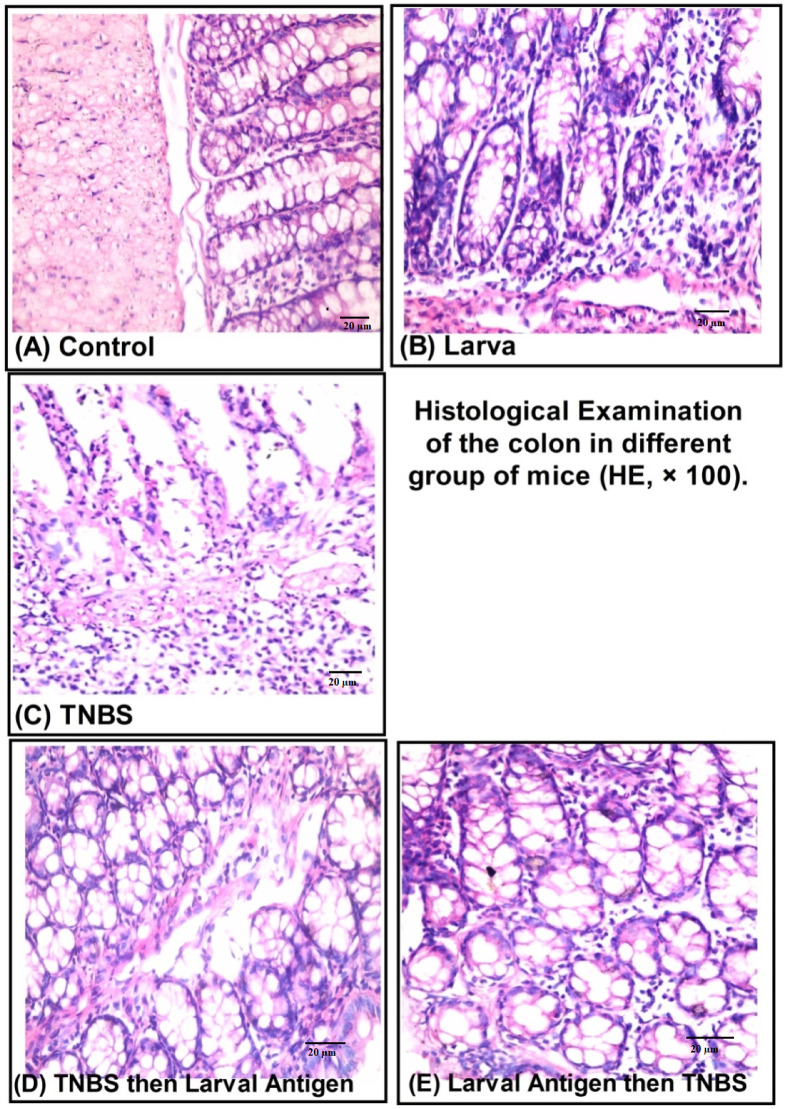
Microscopic picture of H&E sections of mice colons of different groups (*n* = 10/group). (**A**) Vehicle control group: normal mucosal lining with goblet cells with low inflammatory grading; (**B**) Larva group: showing minimal destruction of the surface epithelial cells and moderate inflammatory cell infiltrate; (**C**) TNBS-induced colitis group: showing mucosal edema, hemorrhage, erosions, necrosis with acute inflammatory cell infiltration; (**D**) Treatment (TNBS then larva) group: showing mild erosion of the covering mucosa, moderate inflammatory cell infiltrate of the colonic wall; (**E**) Preventive (Larva then TNBS) group: showing no erosion or ulceration of the covering mucosa, mild inflammatory cell infiltrate of the colonic wall.

**Fig. 7:**
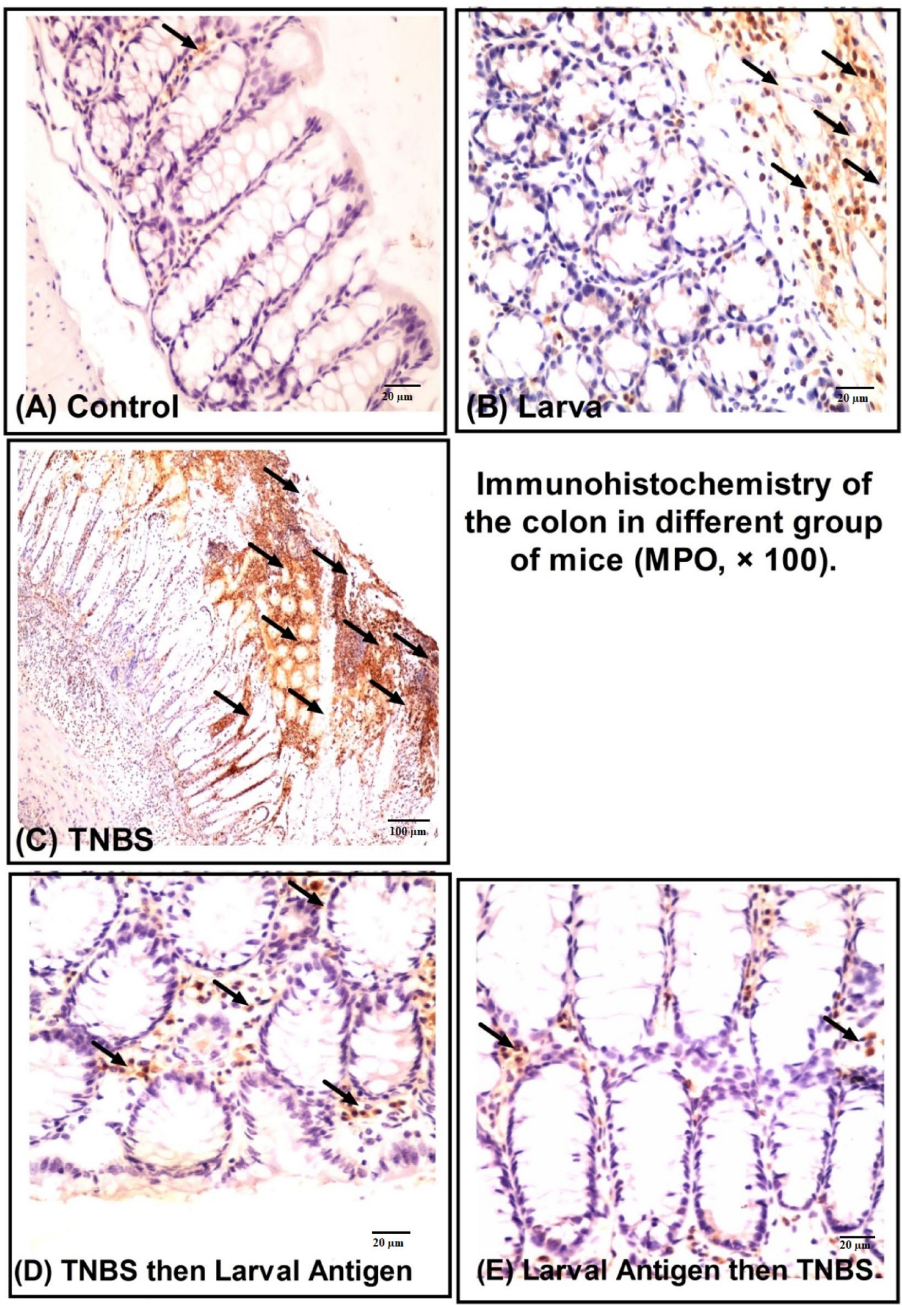
Myeloperoxidase immunostaining of mice colons of different groups (*n* = 10/group). (**A**) Vehicle control group: showing few acute inflammatory cells as demonstrated by positive MPO cytoplasmic staining; (**B**) Larva group showing moderate MPO-positive acute inflammatory cells in the colonic wall; (**C**) TNBS-induced colitis group: showing dense MPO-positive acute inflammatory cells at all layers of the colonic wall ; (**D**) Treatment (TNBS then larva) group: showing mild MPO-positive inflammatory cell infiltrate in the colonic wall and (**E**) Prophylaxsis (larva then TNBS) group: showing moderate MPO-positive acute inflammatory cells in the colonic wall.

As shown in Fig. 6A, the control specimens exhibited normal colonic histological structure. In contrast, TNBS-induced colitis was characterised by marked histopathological alterations, including mucosal oedema, hemorrhagic foci, epithelial erosion, cellular necrosis, and dense acute inflammatory infiltrates. (Fig. 6C). Larva group showed minimal destruction of the surface epithelial cells and moderate inflammatory cell infiltrate (Fig. 6B); Treatment (TNBS then larva extract) group showed mild erosion of the covering mucosa and moderate inflammatory cell infiltrate of the colonic wall (Fig. 6D). The preventive group showed no erosion or ulceration of the covering mucosa with mild inflammatory cell infiltrate of the colonic wall (Fig. 7E).

### Immunohistochemical staining of myeloperoxidase

The disease severity assessments (grading index) revealed distinct patterns of inflammation across experimental groups. The TNBS group demonstrated maximal disease severity (grading index: 36.3 ± 7.6), confirming robust colitis induction, while the Larva group showed intermediate inflammation levels (16.9 ± 1.5). Both therapeutic interventions significantly attenuated disease severity, with the treatment group achieving a 44.9% reduction (20.0 ± 4.3) and the preventive group showing superior protection with a 64.2% reduction (13.0 ± 5.4) relative to TNBS controls (Table 2). MPO immunostaining results similarly revealed intense neutrophil infiltration in TNBS controls (61.5 ± 10.0) (Table 2; Fig. 7C), baseline activity with larval antigen alone (21.4 ± 12.9) (Table 2; Fig. 7B), and significant anti-inflammatory effects from both interventions - the treatment group showed a 71.5% reduction in MPO activity (17.5 ± 3.8) (Table 2; Fig. 7D), while the preventive approach achieved a 52.8% reduction (29.0 ± 21.9) despite showing greater variability in responses (Table 2; Fig. 7E).

**Table 2:** Effects of TNBS-induced colitis and *T. spiralis* larval antigen on average grading index and myeloperoxidase immunostaining in mice.

Group	Control	Larva	TNBS	Treatment	Preventive
Parameter					
Average grading index	4.7 ± 1.2	16.9 ± 1.5	36.3 ± 7.6	20.0 ± 4.3	13.0 ± 5.4
Myeloperoxidase immunostaining	4.1 ± 1.2	21.4 ± 12.9	61.5 ± 10.0	17.5 ± 3.8	29.0 ± 21.9

## Discussion

This study investigated the potential preventive and therapeutic activities of *Trichinella spiralis* larval antigen extract in a murine model of TNBS-induced colitis. The TNBS colitis model was selected based on its well-established validity for investigating inflammatory bowel diseases (IBDs) because it mimics numerous macroscopic and histopathological features observed in human IBD pathology^39^. It is hypothesized that ethanol could disrupt the mucosal barrier, whereas TNBS alone mediates the haptenization of colonic autologous proteins, thereby presenting their immunogenicity to the host immune system. Furthermore, it is a useful model for evaluating T helper cell-mediated immune responses^40^.

Current therapy for IBDs includes the use of aminosalicylate, corticosteroids, various types of immunosuppressive drugs, and even using different antibiotics^41^. Nevertheless, there is an urgent need for novel pharmaceutical agents for IBD treatment to decrease the adverse effects and clinical limitations associated with conventional therapies.

The therapeutic application of helminths or their antigenic components has emerged as a promising research focus in current therapeutic approaches for IBD management^42^. *T. spiralis*-derived antigens express direct immunomodulatory activity on the host immune response through maturation of dendritic cells derived from bone marrow with increased production of a mixture of Th1/Th2 cytokine profiles^21^. Moreover, direct infection with viable *T. spiralis* larvae ameliorated colonic damage in murine models. Such protective effects were mediated by the induction of a Th2-type immune response, as evidenced by the emergence of IL-4 and IL-13 in the supernatants of spleen cell cultures^43^. However, treatment with live helminthes has substantial physical and ethical limitations. Consequently, immunomodulatory proteins secreted by helminths have emerged as more viable therapeutic targets compared to live parasite infection for immunotherapy applications^44^.

In the current study, TNBS-induced colonic lesions were established via different characteristics, including body weight reduction and increased weight-to-length colonic ratio, in addition to histopathological examinations. The prophylactic administration of crude *T. spiralis* larval antigen extract prior to colitis induction significantly attenuated disease severity, as evidenced by reduced weight loss, a normalized colon weight-to-length ratio, and improved macroscopic and histopathological inflammatory scores. Moreover, the administration of larval antigen extract before TNBS induction was associated with a reduction in serum nitrite levels alongside with an elevation in colonic IL-10 levels. Conversely, although treatment with *T. spiralis* antigen ameliorated all pathological features of ulcerative colitis in induced mice, the serum nitrite levels remained high comparable to those in the TNBS alone group, despite a persistent rise of local IL-10 levels.

The current study results indicated a dual role of *T. spiralis* larval antigen. The apparent contradiction, wherein the antigen alone induced mild inflammatory features (elevated NO, MPO, and edema) while ameliorating TNBS-induced colitis can be reconciled through the immunological concept of inflammatory preconditioning^45^. The pro-inflammatory effects observed during monotherapy likely reflect beneficial immune system priming, where antigen exposure triggers transient, low-level activation of innate defenses that subsequently stimulate protective Th2/regulatory responses^46^. In established colitis, this preconditioning enables the same antigen to polarize immune responses toward regulatory pathways (e.g., IL-10 production) that counteract TNBS-driven Th1/Th17 inflammation, consistent with the hygiene hypothesis^47^. These dose- and context-dependent effects^48^ manifest differently across microenvironments: in healthy intestine, the antigen primes immunity without pathology, while in inflamed tissue it promotes resolution through regulatory mechanisms^49^, demonstrating how temporal and spatial factors determine its net immunological impact.

Spleen weight was measured as an indicator of systemic immune activation, as splenomegaly often correlates with inflammation in colitis models^32^. The increase in spleen weight in TNBS-treated mice reflects systemic immune response, while its reduction in the prevention group suggests modulation of systemic inflammation by the larval antigen. This aligns with the observed changes in IL-10 and MPO, supporting the antigen’s systemic immunomodulatory role.

In contrast to the current study, Wang and colleagues reported a significantly stronger therapeutic role for *T*. *spiralis* derivatives than for their protective effects. Their study evaluated both the prophylactic and therapeutic properties of T. *spiralis* adult worm excretory/secretory products (ES) in a murine model of dextran sulfate sodium (DSS)-induced colitis. Treatment with ES products markedly attenuated colitis severity, as evidenced by reduced body weight loss, improved clinical symptoms, lower histological scores, and diminished myeloperoxidase (MPO) activity compared with untreated controls^50^. Similarly, Ma et al. reported that administration of *T*. *spiralis* crude protein alleviated DSS-induced colitis pathology, potentially through Gasdermin-D (GSDMD)-mediated pyroptosis. Furthermore, elevated levels of anti-inflammatory cytokines, including Transforming Growth Factor (TGF-β), IL-4, and IL-10, were observed in the *T*. *spiralis-treated* group, suggesting an immunomodulatory mechanism of action^16^.

According to Xu et al., *T. spiralis* cysteine and serine protease inhibitors exhibited distinct therapeutic and protective effects in a murine model of experimental IBD induced by TNBS. These findings suggest that macrophages may contribute to the mechanism by which recombinant proteins alleviate colitis. IL-33 expression was significantly decreased, whereas IL-6 expression was significantly increased in both therapy and preventive groups^51^.

An earlier study supported the overt protective role of *the T. spiralis* protein when Du et al. evaluated *the T. spiralis* ES protein (53 kDa protein) injected subcutaneously in laboratory mice prior to TNBS induction of colitis. Du and co-workers demonstrated notable reductions in the disease activity index, macroscopic and microscopic inflammation scores. At the same time, in contrast to the present study, the authors detected a decline in IL-10 levels^29^. Similarly, IL-10 transgene supplementation was successful in IL10-/- mice, as evidenced by the serum IL-10 level, and significantly reduced index scores of enterocolitis activity, increased weight-to-length colonic ratios, and decreased scores of microscopic inflammation^52^. Moreover, intravenous delivery of an adenoviral vector encoding IL-10 reversed the pathology of TNBS-induced colitis^53^.

Zheng et al. investigated the optimal exposure time for *T*. *spiralis* derivatives promoting protective effects by comparing pre-exposure of mice to *T. spiralis* 3 weeks before induction and immediately after (DSS)-induced colitis. The study revealed that prior exposure to *T. spiralis* did not exert protective effects compared with the introduction of the parasite during the acute phase of DSS-induced colitis. Additionally, their findings suggested that the impending mechanisms of action likely involve a synergistic interaction between IL-17 and TNF-alpha, along with an immunosuppressive role^54^.

Likewise, Gunasekera and colleagues have demonstrated the mechanistic relationship between IL-10 and IL-22, suggesting important insights into the pathogenesis of chronic colitis in IL-10^−/−^ mice. Their findings highlight the critical role of the IL-10/IL-22 axis in establishing a regulatory network within the gut mucosa, which is vital for maintaining intestinal homeostasis and promoting the resolution of intestinal inflammation^8^.

Pils et al. established that genetic inactivation of the IL-10 receptor in mice heightens susceptibility to chemically induced colitis. Their study further outlined the cellular targets of IL-10-mediated immune regulation, revealing that suppression of the immune response depends on IL-10 receptor expression in monocytes/macrophages and/or neutrophils, but not in T cells or B cells^55^. TGF-β, IL-4, IL-13, and IL-10 levels were elevated, whereas IL-6 and IFN-γ were significantly diminished in isolated splenic lymphocytes in laboratory animals with colitis induced by DSS next following the treatment with ES products from *T. spiralis* adult worms^56^. Moreover, IL-10-deficient mice exhibit marked elevation in neutrophils and splenic macrophages, which is suppressed by IL-10 signalling specifically in myeloid cells, with no observed effect in B cells^57^.

To evaluate neutrophil involvement, neutrophil infiltration was analyzed using myeloperoxidase (MPO) immunostaining^58^. The results showed a reduction in MPO expression when *T. spiralis* larval antigen extract was administered both prior to and subsequent to TNBS-induced colitis. The breaking of leukocyte infiltration has been suggested as a key mechanism underlying the protective role of IL-10 in colitis^59^. Conversely, leukocytes, for example, macrophages and neutrophils, in human intestinal tissue lead to the overproduction of IL-10 independently of T cells^60,61^.

The regulatory role of IL-10 in nitric oxide (NO) production remains incompletely understood. To investigate the interplay between endogenous IL-10 and nitrite (a stable end product of NO) in experimental colitis, studies were conducted using IL-10/iNOS double-knockout and IL-10 single knockout mice. After 3–4 months, animals with inducible nitric oxide synthase (iNOS) developed mucosal damage, granulocyte infiltration, and elevated iNOS mRNA and protein expression in intestinal tissues. Notably, iNOS⁻ and ⁻/IL-10⁻/⁻ mice exhibited pathological features similar to IL-10⁻/⁻ mice, including abnormal crypt architecture, depletion of goblet cells, intestinal wall thickening, and granulocyte infiltration. These findings suggest that iNOS does not significantly influence the progression or severity of spontaneous chronic inflammation in IL-10-deficient mice^62^. Interestingly, iNOS, a key molecule produced by proinflammatory macrophages^63^, has been implicated in colonic injury, as demonstrated in a previous study^64^. While IL-10 potently inhibited iNOS, and thereby NO production^65^. This study further supports this inverse relationship between IL-10 and NO synthesis, highlighting IL-10’s role as a critical modulator of inflammatory responses in colitis.

Emerging evidence suggests that *T. spiralis* larval antigen exerts its protective effects not restricted to immunomodulation; it may also act through changing microbiome-mediated immunomodulatory mechanisms of induced colitis in mice. Sun et al. demonstrated that helminth infection restructures the gut microbiota, particularly enriching short-chain fatty acid-producing bacteria, which correlates with enhanced goblet cell differentiation, strengthened mucosal barrier function, and elevated anti-inflammatory IL-10 responses^66^.

Complementing these findings, Long and coworkers showed in DSS- and *Salmonella*-induced colitis models that a *T. spiralis*-derived serine protease inhibitor attenuated inflammation by immune modulation via reduced TNF-α and neutrophil infiltration alongside increased IL-10 and M2 macrophage polarization, as well as microbiome restoration evidenced by increased microbial diversity, probiotic abundance, and epithelial barrier integrity^67^. The last two studies highlight the multifaceted therapeutic potential of *T. spiralis* antigens, targeting both host immunity and microbial ecosystems to ameliorate colitis, which needs more future studies^66,67^.

## Conclusion

Our findings support the beneficial preventive and therapeutic role of products derived from *T. spiralis* in alleviating inflammatory colitis instead of infection with live parasites, which is widely intolerable ethically and physically, with possible subsequent pathology. The present data also highlighted the potential role of Interleukin-10 (IL-10) in experimental colitis, with significant correlations with nitric oxide levels in the protective pattern of *T. spiralis* larval antigen extract.

### Limitations of the study

The study outcomes restricted by limited biochemical and histobiochemical markers were investigated to reach definite underlying mechanisms of both the protective and therapeutic roles of the *T. spiralis* larval antigen. Consequently, more, larger studies are necessary to validate these findings and explore the potential advantages of *the T. spiralis* larval antigen.

## Data Availability

All data generated or analysed during this study are included in this published article.
